# The First Appearance of Man on Our Planet

**Published:** 1867-04

**Authors:** 


THE FIRST APPEARANCE OF MAN ON OUR PLANET.—"Although perhaps more interesting in an ethnological point of view, we cannot altogether exclude from our notice the phenomena attending the first appearance of Man on our planet. The discoveries of the last few years have satisfactorily shown that the opinions formerly entertained of a great break existing between the period when the now extinct races of Mammalia dwelt in our land, and the first creation of man, are no longer tenable. Here also we have been obliged to give up the theory of great breaks between successive formations. As we find a gradual passage from one geological formation to another evidenced by the *gradual* dying out of the pre-existing forms of animal life, and the *gradual* introduction of newer, and generally higher forms, (although we do not yet understand the law of such progessive changes,] so, when we come to the most recent, or Qarternary, periods in geological chronology, we find evidence of Man's existence on the earth before the final disappearance of those varied forms of mammalian life which have hitherto been generally looked upon as belonging to the final period of the geological cicle. Thus Man of the present day is connected by an almost unbroken series of links with the recently discovered Foraminifera of the Laurentian gneiss."—*Anniversary Address of the President (Sir R. I. Marchison) of the Geological Society of London*, 1866.

